# Copper Dependent Modulation of α-Synuclein Phosphorylation in Differentiated SHSY5Y Neuroblastoma Cells

**DOI:** 10.3390/ijms22042038

**Published:** 2021-02-18

**Authors:** Marco Greco, Chiara Carmela Spinelli, Lidia De Riccardis, Alessandro Buccolieri, Simona Di Giulio, Debora Musarò, Claudia Pagano, Daniela Manno, Michele Maffia

**Affiliations:** 1 Department of Mathematics and Physics “E. De Giorgi”, University of Salento, 73100 Lecce, Italy; marco.greco@unisalento.it (M.G.); daniela.manno@unisalento.it (D.M.); 2 Department of Biological and Environmental Science and Technology, University of Salento, 73100 Lecce, Italy; chiaracarmela.spinelli@unisalento.it (C.C.S.); lidia.dericcardis@unisalento.it (L.D.R.); alessandro.buccolieri@unisalento.it (A.B.); simona.digiulio@studenti.unisalento.it (S.D.G.); debora.musaro@studenti.unisalento.it (D.M.); claudia.pagano@studenti.unisalento.it (C.P.)

**Keywords:** copper, α-synuclein, Parkinson’s disease (PD), oxidative stress, ROS

## Abstract

Copper (Cu) dyshomeostasis plays a pivotal role in several neuropathologies, such as Parkinson’s disease (PD). Metal accumulation in the central nervous system (CNS) could result in loss-of-function of proteins involved in Cu metabolism and redox cycling, generating reactive oxygen species (ROS). Moreover, neurodegenerative disorders imply the presence of an excess of misfolded proteins known to lead to neuronal damage. In PD, Cu accumulates in the brain, binds α-synuclein, and initiates its aggregation. We assessed the correlation between neuronal differentiation, Cu homeostasis regulation, and α-synuclein phosphorylation. At this purpose, we used differentiated SHSY5Y neuroblastoma cells to reproduce some of the characteristics of the dopaminergic neurons. Here, we reported that differentiated cells expressed a significantly higher amount of a copper transporter protein 1 (CTR1), increasing the copper uptake. Cells also showed a significantly more phosphorylated form of α-synuclein, further increased by copper treatment, without modifications in α-synuclein levels. This effect depended on the upregulation of the polo-like kinase 2 (PLK2), whereas the levels of the relative protein phosphatase 2A (PP2A) remained unvaried. No changes in the oxidative state of the cells were identified. The Cu dependent alteration of α-synuclein phosphorylation pattern might potentially offer new opportunities for clinical intervention.

## 1. Introduction

Parkinson’s disease (PD) is a neurological condition, strongly correlated with aging, with a prevalence of about 0.3% until the age of 60, to over 3% after the age of 80 [1,2]. The disease is most commonly associated with the male gender and several studies linked its onset to a prolonged temporal exposure to industrial chemicals and pollutants, such as pesticides and metals [3,4,5].

PD shows characteristic symptomatology, accompanying the alteration of the capacity to coordinate voluntary movements due to the loss of dopaminergic neurons of the substantia nigra pars compacta [6]. These symptoms are commonly: tremor, stiffness, bradykinesia, and imbalance disturbances [7].

At a cellular level, the pathological hallmarks of PD are characterized by protein aggregates, defined Lewy bodies (LBs), whose main component is represented by the small protein α-synuclein in a phosphorylated and polyubiquitinated form [8].

The phosphorylation and ubiquitination processes are the main strategies to route the protein towards degradation pathways; its accumulation under these forms reinforces the hypothesis of an alteration of these processes as triggers for the disease [9]. Several studies have observed that copper-induced toxic effects, together with increasing levels of α-synuclein in the cell, could hamper degradation pathways and, in particular, the ubiquitin proteasome system (UPS) [10,11].

In humans, copper is one of the most important biometals for the magnitude of the metabolic processes in which it is involved [12]. 

Copper, as a transition metal, exists both in monovalent Cu(I) and in divalent Cu(II) forms, acting as catalytic and structural elements in proteins and other biomolecules. More importantly, organisms have exploited its ability to undergo the redox processes to carry out crucial functions, such as electron transport and cellular respiration [13,14].

A strict control of the copper concentrations in free form is imperative in the organism, since its absorption at the intestinal level. Indeed, an unregulated redox cycling can be deleterious to the cells, leading to harmful effects of reactive oxygen species (ROS) [15]. For this reason, the physiological amount of free copper into the cell is lower than 10^−18^ M; bioavailable copper is detectable in low amounts in the cerebrospinal fluid and in the brain extracellular space, with concentrations of ~70 and ~1 μM, respectively [16].

In the brain, as in other organs, copper is handled by a series of molecules, many of them are moonlighting proteins [17]. Copper enters the cell mainly in a monovalent form through the copper transporter protein 1 (CTR1) transporter, although many evidences also support the involvement of the copper transporter protein 2 (CTR2) and the divalent metal transporter 1 (Dmt1)—the latter conveying its divalent form [18]. Bound in the cytosol by glutathione, it is sorted to specific chaperones, and from those to various intracellular components. Copper transport protein ATOX1 (Atox1) is responsible for transporting the metal to the copper-transporting ATPases 1 (ATP7A) and 2 (ATP7B), or to the extracellular superoxide dismutase [Cu-Zn] (SOD3) protein. ATP7A represents the entry of the secretory pathway; from here, the metal is incorporated into newly synthesized cuproproteins or excreted when present in too high concentrations [19].

Transit of copper to the mitochondria, primarily for the incorporation into the cytochrome c oxidase (COX), is mediated by the cytochrome c oxidase copper chaperone (Cox17), cytochrome c oxidase assembly protein 1 (Sco1) and 2 (Sco2) chaperones. The function of the copper chaperone for superoxide dismutase (CCS) is also essential in the cell; it provides metal to the superoxide dismutase [Cu-Zn] (SOD1), one of the main eukaryotic antioxidant systems. Lastly, the cell is able to maintain a certain amount of copper retained with reserve functions carried out through proteins with high binding affinity, such as the metallothioneins [20,21].

In CNS, copper performs many peculiar functions, in addition to those common to the other body districts. It is involved in catecholamine synthesis and degradation, neuropeptide synthesis, glutamatergic synapse modulation, excitotoxicity, myelin formation, and it also works as a neurotransmitter [22,23,24].

Alterations in the copper homeostatic mechanisms are deeply related to the neurodegeneration processes. Copper increase in neurons can cause the paired reactions of Fenton and Haber–Weiss, involved in the conversion of the superoxide anion and hydrogen peroxide into the highly reactive hydroxyl radical species [12]. 

The pathological scenario observed in the claimed PD is well-documented, and often associated with oxidative stress, mitochondrial dysfunction, and, sometimes, loss of function of the “sequestered” proteins. To date, however, there are no clear indications on what happens in neural cells during the early stages of the disease development. Furthermore, we have to consider the diverse etiological factors for the multiple PD forms.

The difficulty in studying these processes is given by the multifactorial nature and by the complex level of interactions existing among the different cell types in the CNS. Moreover, researchers have to face the difficulty of reproducing the physiological characteristics of a human dopaminergic neuron in vitro; at the moment, there are no models currently available, alone, to provide complete information on the onset of the disease [25].

Our study was based on the use of the human neuroblastoma SHSY5Y cell line, often used as an in vitro model for testing the effects of neurotoxins suspected of being associated with the PD outbreak.

These cells possess dopaminergic features, including the tyrosine hydroxylase (TH) and the dopamine transporter (DAT) expression, even if at low levels [26]. Moreover, as cancerous cells, they can be expanded, virtually indefinitely, to generate the necessary number of cells required for the experiments. The disadvantage of continuous mitosis, which is poorly suitable for studying non-divisible cells as neurons, is overcome by a process of differentiation.

The differentiated SHSY5Y cells acquire, moreover, both phenotypic and genotypic characteristics closer to those of the dopaminergic neurons. Their treatment with compounds believed to artificially induce the onset of PD—mimicking events, such as the alteration of mitochondrial homeostasis and the increase in oxidative stress and protein degradation processes—allowed us to observe cellular responses rather similar to those expected in vivo, and the limitations due to the in vitro conditions.

## 2. Results

**Figure 1:**
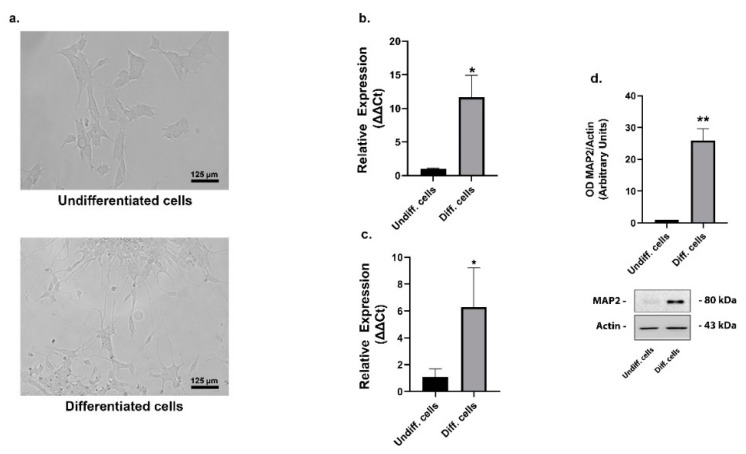
SHSY5Y cells differentiation. Representative phase contrast images of cellular morphology of SHSY5Y cells before (top left) and after (bottom left) the differentiation protocol. Morphology of differentiated cells showed prominent neurites outgrowth (**a**). Relative expression of the neuronal markers TH mRNA (**b**) and synaptophysin (**c**) significantly increased in differentiated cells (means ± SD of three independent experiments). Representative immunoblots of the neuronal marker MAP2 (**d**) in undifferentiated and differentiated cells. Densitometric analysis of bands represented means ± SD of three independent experiments (right). β-actin was used as loading control (**c**). The results were presented as means ± standard deviation of three independent experiments; values for differentiated cells were compared to undifferentiated cells by one-way ANOVA following Tukey test * *p* < 0.0332 ** *p* < 0.0021.

The human neuroblastoma SHSY5Y cells were differentiated by a sequential handling with retinoic acid (RA), followed by the brain-derived neurotrophic factor (BDNF) and B27 treatments. The RA, together with the low serum levels in the medium, induced the interruption of the cell cycle and the initial differentiation events; the subsequent BDNF/B27 treatment, in the total absence of serum, allowed the formation of a homogeneous population of neuron-like cells [27]. Figure 1a shows the changes in morphological features of differentiated SHSY5Y compared with undifferentiated ones. Differentiated cells had some long neuritic processes emerging from the cell body, coherent with increased expression of the microtubule-associated protein 2 (MAP2), a neuronal cytoskeletal marker (Figure 1d). Moreover, cell differentiation was verified by the mRNA level increase of the neuronal markers, TH and synaptophysin (Figure 1b,c).

The cells were treated with increasing concentrations of copper chloride (1–100 µM) and rotenone (1–10 µM) for 24 and 48 h, and the influence of treatments on their viability was assayed by using MTT test. These ranges were chosen on the basis of previous studies on the copper and rotenone toxicity in SHSY5Y and in other neuronal cells [28,29,30,31]. The use of rotenone was considered, given its well-known inhibitory activity toward the mitochondrial transport chain. The purpose was to evaluate the extent of oxidative stress and the cytotoxic effect of copper compared to that of a well-known oxidizing molecule, also known to be associated with the onset of neurodegenerative diseases [32].

**Figure 2:**
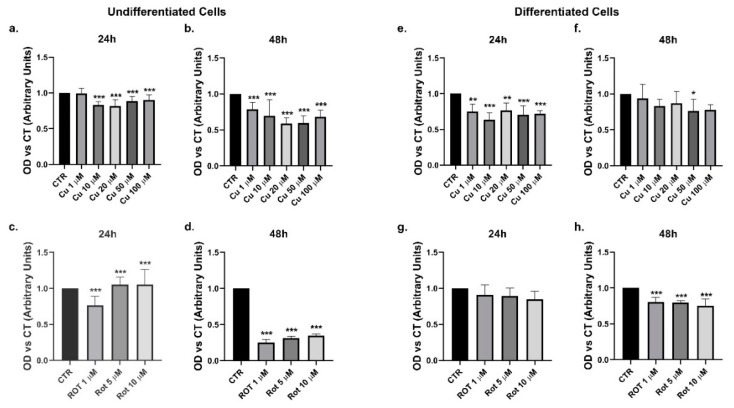
Effects of copper on cell viability (relative MTT assay). Undifferentiated SHSY5Y cells were treated with the indicated concentrations of copper or rotenone for 24 h (**a**,**c**) and 48 h (**b**,**d**). The same treatments were performed in differentiated cells, also in this case for 24 h (**e**–**g**) and 48 h (**f**–**h**). The results are presented as means ± SD of seven replicates; values were compared with untreated cells (CTR) by one-way ANOVA following Tukey test * *p* < 0.0332 ** *p* < 0.0021 *** *p* < 0.0002, compared to untreated cells (CTR).

A copper dependent decrease of the cell viability occurred when the extracellular metal concentrations changed from 1 to 20 µM; it did not modify significantly at 50 and 100 µM (Figure 2a). The effect was most pronounced after 48 h of treatment, with a dose dependent viability reduction from 1 to 50 µM (Figure 2b). Rotenone was not toxic to cells under any of the conditions tested after 24 h (Figure 2c). On the other hand, the cell viability reduced by 70% at a rotenone concentration of 1 µM and remained so at 5 and 10 µM (Figure 2d). Overall, the differentiated cells appeared to be more resistant to both copper (Figure 2e,f) and rotenone (Figure 2g,h) treatments and the viability did not change between 24 and 48 h. Results were in line with literature, as rotenone—a classic inhibitor of mitochondrial complex I—was known to be toxic to several cell lines [33]. To maintain the homogeneity of treatments, we incubated the cells with 20 or 50 µM copper for 48 h, and with 5 or 10 µM rotenone for 24 h.

**Figure 3:**
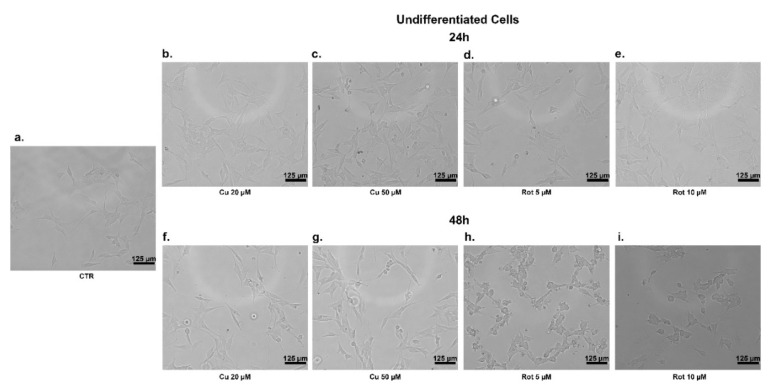
SHSY5Y undifferentiated cells treatments. Phase contrast images of cellular morphology of SHSY5Y undifferentiated cells after copper (**b**,**c**,**f**,**g**) and rotenone (**d**,**e**,**h**,**i**) treatments for 24 and 48 h. The morphological alteration in cells treated with rotenone for 48 h is noticeable, especially compared with the untreated cells on the left (**a**).

**Figure 4:**
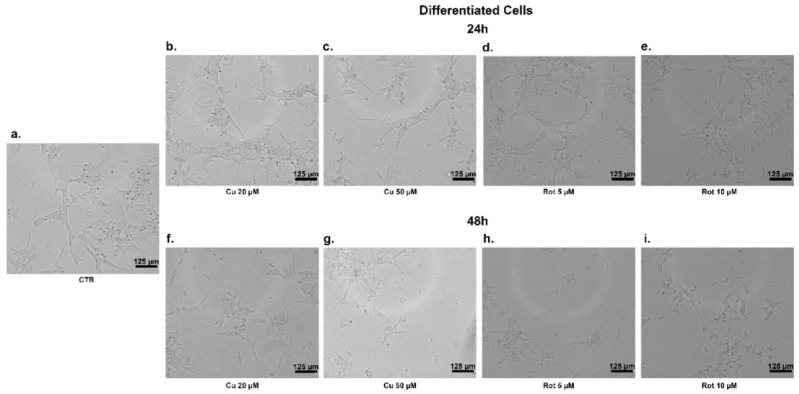
SHSY5Y differentiated cells treatments. Phase contrast images of cellular morphology of SHSY5Y differentiated cells after copper (**b**,**c**,**f**,**g**) and rotenone (**d**,**e**,**h**,**i**) treatments for 24 and 48 h. Untreated cells on the left (**a**).

In the undifferentiated cells, at 24 h, neither copper nor rotenone significantly affected their morphology and viability (Figure 3b–e and Figure 4b–e); similarly, 48 h of copper treatment caused no change in cells shape (Figure 3f,g) compared to untreated ones (Figure 3a). At 48 h of treatment with rotenone, we observed that the SHSY5Y cells changed their form, becoming rounder, partially detached from monolayers, and floated in the medium (Figure 3h,i). No morphological alterations to differentiated cells were evident in any of the conditions considered (Figure 4a–i).

**Figure 5:**
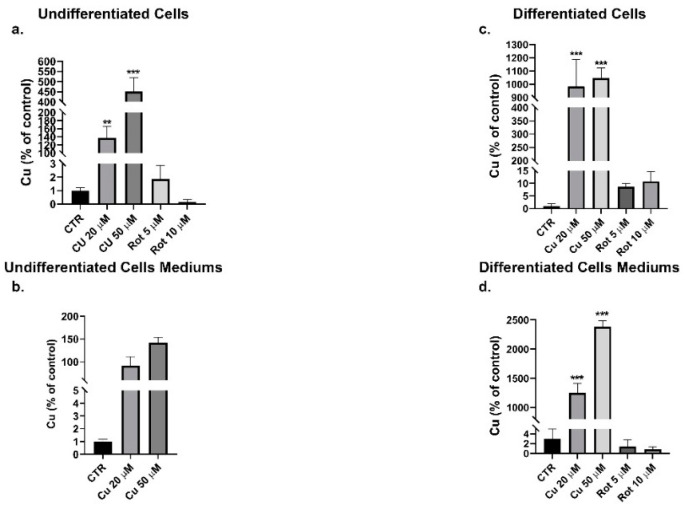
Atomic absorption spectrometry. Undifferentiated (left) and differentiated (right) cells treated with the indicated concentrations of copper for 48 h or rotenone for 24 h were analyzed by atomic absorption spectrometry to determine the intracellular copper content (**a**–**c**) and the residual content of the medium (**b**–**d**) respectively. Histogram bars represent means ± SD of three independent experiments. Values of intracellular copper were normalized on total protein and compared with untreated cells (CTR) by one-way ANOVA following Tukey test ** *p* < 0.0021 *** *p* < 0.0002.

Following exposure of the undifferentiated and differentiated cells to copper or rotenone, the metal content in intracellular (Figure 5a,c) and extracellular media (Figure 5b,d) was determined by the atomic absorption spectrometry. The intracellular metal concentration increased significantly, in a dose-dependent manner, after extracellular copper incubation (Figure 5a). To note, the copper uptake was greater in the differentiated SHSY5Y (Figure 5c), to confirm their increased metal demand, probably due to the presence of copper-dependent enzymes involved in neurotransmitter production [34].

**Figure 6:**
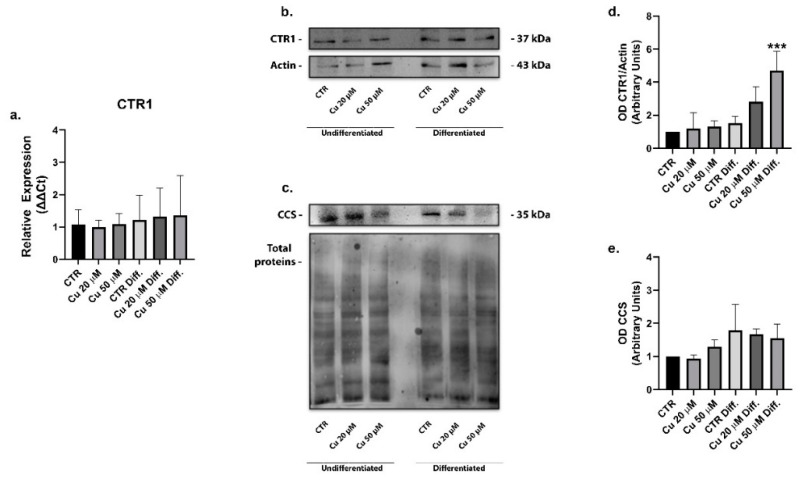
Copper treatment increased copper transporter protein 1 (CTR1) expression in differentiated SHSY5Y cells. mRNA relative expression of the transporter CTR1 in undifferentiated and differentiated cells incubated with 20 or 50 µM copper (**a**). Representative immunoblots of the CTR1 (**b**) and copper chaperone for superoxide dismutase (CCS) (**c**) protein levels and densitometric analysis of bands representing means ± SD, respectively, of five (**d**) and three (**e**) independent experiments. β-actin was used as loading control for CTR1 (**b**), CCS levels were normalized on total protein (c). Values were compared with untreated cells (CTR) by one-way ANOVA following Tukey test *** *p* < 0.0002.

Intracellular copper uptake is primarily mediated by the CTR1, an evolutionarily conserved transporter acting ubiquitously in all tissues [35]. The expression of CTR1 was analyzed in the undifferentiated and differentiated cells treated with 20 or 50 µM copper for 48 h. While no significant changes in mRNA levels were observed (Figure 6a), the protein was increased 1.6-fold upon differentiation and of 2.9- and 4.8-fold after incubation with 20 or 50 µM copper, respectively, in comparison with the undifferentiated SHSY5Y (Figure 6b,d). The increase observed in differentiated cells following treatment with 50 µM copper is significant. Therefore, the differentiation process and copper supplementation induced a significant increase of the CTR1 protein expression.

Once in the cells, copper is delivered to the specific destinations by small soluble proteins called copper chaperones [36]. The cytosolic chaperone copper chaperone for superoxide dismutase (CCS) escorts copper to SOD1, a copper-dependent superoxide dismutase responsible for ROS detoxification. In our study, the CCS protein expression did not change after copper treatments (Figure 6c,e), in accordance with formerly obtained data showing no difference in CCS mRNA levels between the undifferentiated and differentiated SHSY5Y cells [34].

CCS represents an indicator of copper cell tolerance, with its levels decreasing in the presence of metal overloads in order to prioritize its removal. Its unaltered levels suggested that both the differentiated and non-differentiated SHSY5Y cells were able to effectively handle the high copper intakes.

**Figure 7:**
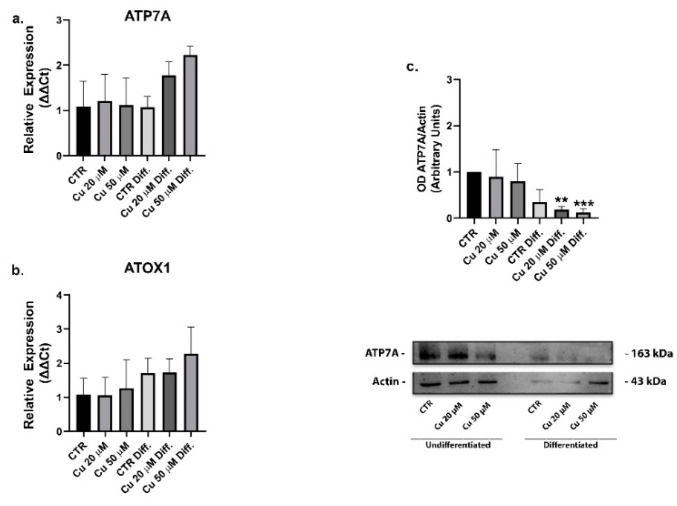
Copper treatment did not change the expression of the chaperone Atox1 and ATP7A transporter. mRNA relative expression of the transporter ATP7A (**a**) and Atox1 chaperone (**b**) in undifferentiated and differentiated cells incubated with 20 or 50 µM copper. Representative immunoblots of the ATP7A protein and densitometric analysis of bands representing means ± SD of three independent experiments (**c**). β-actin was used as loading control. Values were compared with untreated cells (CTR) by one-way ANOVA following Tukey test ** *p* < 0.0021 *** *p* < 0.0002.

The cytosol chaperone Atox1 shuttles copper to the ATP7A transporter that, by hydrolyzing ATP, can translocate copper toward the trans-Golgi network to support the metalation of emerging apoproteins, the activation of copper-dependent enzymes or the removal of copper excess from the cell. Altogether, this is defined as secretory pathway [36,37]. Although it was known that the ATP7A levels in differentiating cells were increased in response to RA, but not BDNF [34], the supplemental copper could be sent to the secretory pathway. On the contrary, the mRNA levels of Atox1 and ATP7A (Figure 7a,b) did not vary significantly in our experimental conditions and the western blot analyses highlighted a significative reduction of protein ATP7A in the differentiated and copper treated cells (Figure 7c). The gene encoding for ATP7A was known to be strongly responsive to the RA β2 receptor; this finding could explain the high levels of the transporter described in the literature after RA treatments. The differentiation we carried out, foreseeing a final step without RA, allowed a return of the protein expression to more physiological levels [38].

**Figure 8:**
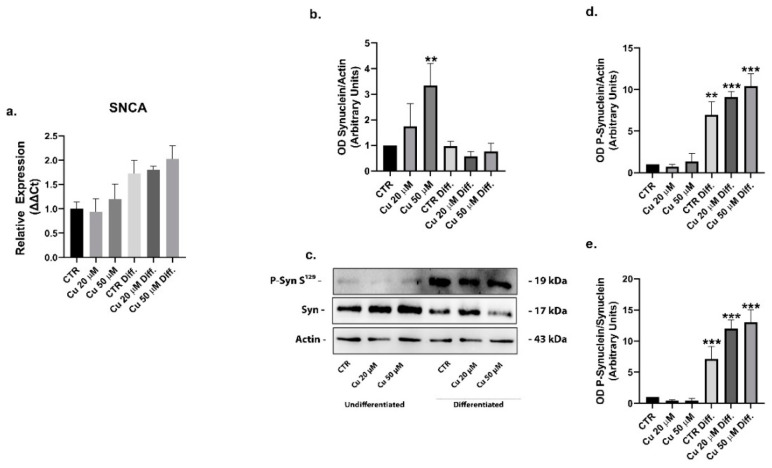
Copper treatment increased phospho-α-synuclein levels in differentiated SHSY5Y cells. mRNA relative expression of α-synuclein gene (SNCA) in undifferentiated and differentiated cells incubated with 20 or 50 µM copper (**a**). Representative immunoblots of the α-synuclein and phospho-α-synuclein protein (**c**) and densitometric analysis of bands representing means ± SD of three independent experiments (**b**,**d**,**e**). β-actin was used as loading control. Values were compared with untreated cells (CTR) by one-way ANOVA following Tukey test ** *p* < 0.0021 *** *p* < 0.0002.

The interaction of metals with α-synuclein has been shown to trigger its phosphorylation and aggregation. The protein was upregulated in the undifferentiated SHSY5Y cells treated with 50 µM copper, while the phosphorylated (S129) α-synuclein expression was not changed (Figure 8b–e). After the SHSY5Y cells differentiation, copper incubation did not alter the total expression of α-synuclein (Figure 8a) but, surprisingly, the phosphorylation of the protein was significantly increased after differentiation: 6.9- and 9-fold in untreated cells and in cells treated with 20 µM copper concentrations, respectively. The maximum level of phosphorylated protein was observed after 50 µM copper treatments, 10.4 times higher with respect to undifferentiated cells (Figure 8b–e).

**Figure 9:**
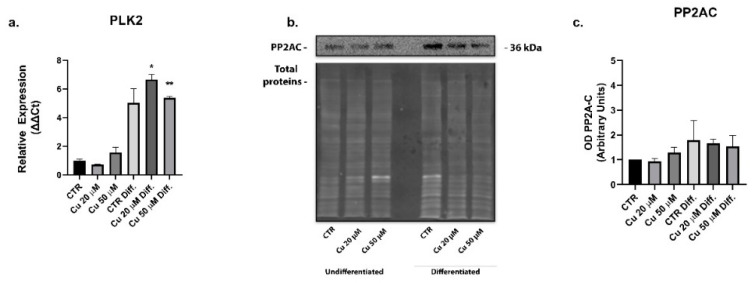
Copper treatment induced the expression of the kinase PLK2 in differentiated SHSY5Y cells. mRNA relative expression of PLK2 in undifferentiated and differentiated cells incubated with 20 or 50 µM copper (**a**). Representative immunoblots of the phosphatase PP2A (**b**) and densitometric analysis of bands representing means ± SD of three independent experiments (**c**). PP2A values were normalized on total proteins. Values were compared with untreated cells (CTR) by one-way ANOVA following Tukey test * *p* < 0.0332 ** *p* < 0.0021.

To justify this result, the cellular expressions of PLK2 kinase, involved in phosphorylation at the residue S129 of α-synuclein and the relative phosphatase PP2A, were analyzed. PP2A western blots did not show a significant variation between the different conditions (Figure 9b,c), whereas the expression of PLK2 was upregulated in both the untreated and copper treated differentiated cells (Figure 9a).

Together, these results suggested that the higher phosphorylation of α-synuclein in the differentiated cells could be mediated by the PLK2 kinase.

**Figure 10:**
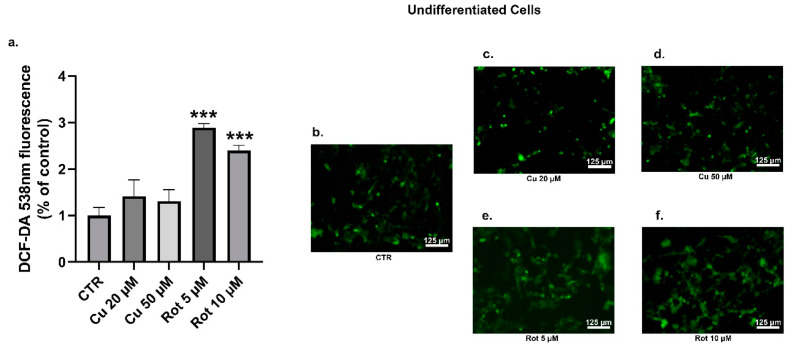
Undifferentiated SHSY5Y cells DCFDA analysis. The relative intensity of DCF was employed to evaluate the reactive oxygen species (ROS) levels. Cells were treated with 20 or 50 µM copper (**c**,**d**) or 5 or 10 µM rotenone (**e**,**f**) and compared to untreated cells (**b**). Fluorescence values were normalized on total protein (**a**). Values were compared with untreated cells (CTR) by one-way ANOVA following Tukey test *** *p* < 0.0002.

**Figure 11:**
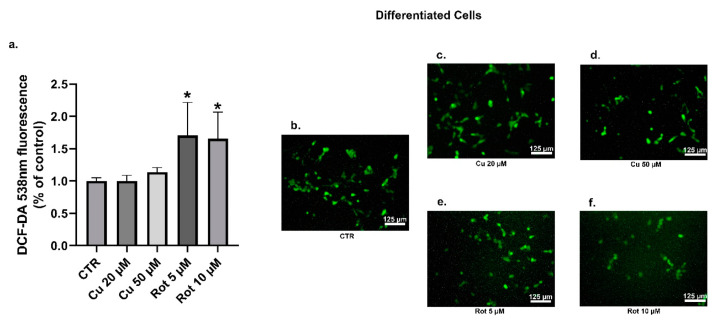
Differentiated SHSY5Y cells DCFDA analysis. The relative intensity of DCF was employed to evaluate the ROS levels. Cells were treated with 20 or 50 µM copper (**c**,**d**) or 5 or 10 µM rotenone (**e**,**f**) and compared to untreated cells (**b**). Fluorescence values were normalized on total protein (**a**). Fluorescence values were normalized on total protein (a). Values were compared with untreated cells (CTR) by one-way ANOVA following Tukey test * *p* < 0.0332.

The oxidative stress has been implicated in the propagation of neuronal dysfunction in multiple neurodegenerative diseases. In addition, the increased ROS production was also related to the phosphorylation of α-synuclein [39,40]. In order to evaluate the features of the oxidative stress induced by both copper and rotenone, the levels of intracellular ROS were examined through a DCFDA assay. No significant changes in the ROS levels were detected between the undifferentiated (Figure 10a–d) and differentiated cells (Figure 11a–d) treated with copper. As expected, endogenous ROS production was significantly increased in the SHSY5Y cells treated with rotenone, when compared to the untreated condition (Figure 10a,b,e,f and Figure 11a,b,e,f).

**Figure 12:**
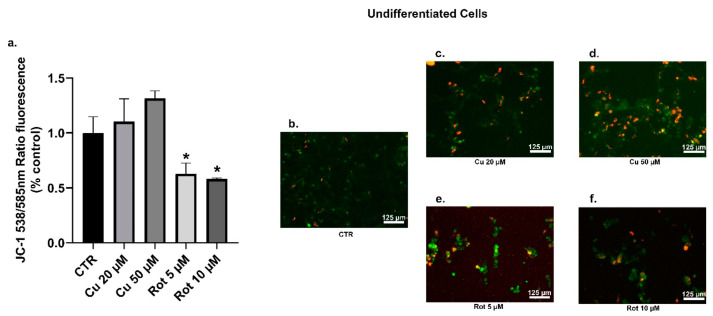
Undifferentiated SHSY5Y cells JC-1 analysis. The JC-1 staining was used to assess the mitochondrial membrane potential (MMP) cells were treated with 20 or 50 µM copper (**c**,**d**) or 5 or 10 µM rotenone (**e**,**f**) and compared to untreated cells (**b**). The percentages of JC-1 monomers presented the percentages of SHSY5Y cells with low MMP. Fluorescence values were normalized on total protein (**a**). Values were compared with untreated cells (CTR) by one-way ANOVA following Tukey test * *p* < 0.0332.

**Figure 13:**
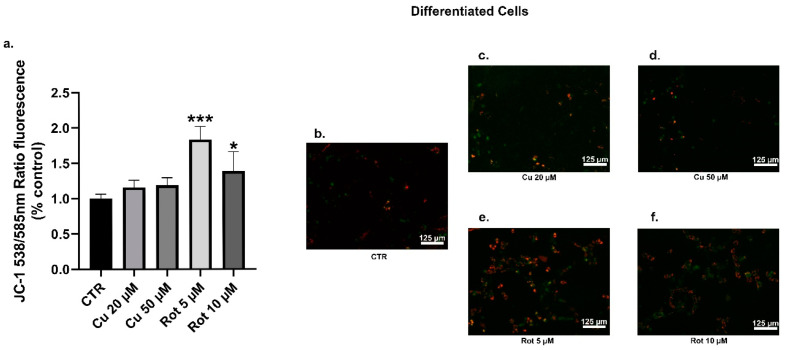
Differentiated SHSY5Y cells JC-1 analysis. The JC-1 staining was used to assess the MMP. Cells were treated with 20 or 50 µM copper (**c**,**d**) or 5 or 10 µM rotenone (**e**,**f**) and compared to untreated cells (**b**). The percentages of JC-1 monomers presented the percentages of SHSY5Y cells with low MMP. Fluorescence values were normalized on total protein (**a**). Values were compared with untreated cells (CTR) by one-way ANOVA following Tukey test * *p* < 0.0332 *** *p* < 0.0002.

The mitochondria are one of the most important sources of cellular ROS; so, we examined the effects of copper and rotenone on the mitochondrial membrane potential (MMP) by using the cell permeable lipophilic fluorescent JC-1 dye (Figure 12 and Figure 13). Our data showed that the exposure of undifferentiated cells to copper led to a moderate and non-significant dose-dependent increase in the MMP (Figure 12a–d), whereas no changes were observed in the differentiated cells (Figure 13a–d). As we expected, rotenone, both at 5 and 10 μM concentrations, caused significant membrane depolarization in the undifferentiated cells (Figure 12a,e,f).

The significant mitochondrial membrane hyperpolarization observed in the differentiated cells (Figure 13e,f) could be explained, according to the literature, by a RA and BDNF-dependent formation of the mitochondrial reserve potential during the differentiation processes [41]. This finding was particularly evident after rotenone treatments that could induce ATP-dependent repolarization processes involving the nucleotide translocase adenine (ANT) or the Complex V of the mitochondrial electron transport chain [42].

The rotenone activity could be explained by its decupling action in the mitochondrial electron transport chain and its capability to inhibit mitochondrial complex I. This led to an irreversible formation of a large membrane permeability transition (MPT) pore on the inner mitochondrial membrane, which allowed the influx and efflux of ions and molecules, as well as further dissipation of the MMP [43,44,45]. The swelling and eventually rupture of the outer mitochondrial membrane caused the release of calcium and proapoptotic proteins, such as cytochrome c from the organelle [46].

## 3. Discussion

The α-synuclein protein is a key regulator of PD pathology as it is the manly component of LBs. Phosphorylation at the residue Serine 129 (S129) is of a particular interest because the majority of synuclein in LBs contains this modification. Although α-synuclein is constitutively phosphorylated at low levels in normal brains [47,48], an abnormal accumulation of S129 phosphorylation is found in the synucleinopathies [49,50]. Transition metals have been implicated in the PD pathogenesis. Particularly, copper binds with a high affinity at two sites of α-synuclein protein and mediates fibrillation and aggregation process [51,52,53,54]. The role of copper and all mechanisms correlated to α-synuclein accumulation and toxicity are sufficiently understood. In the present study, we evaluated the relationship among neuronal differentiation, Cu, and α-synuclein. We used SHSY5Y human neuroblastoma cell line, as an in vitro model widely used in PD research, and we differentiated them into neuronal-like cells to reproduce some of the dopaminergic neuron characteristics affected by the disease.

The atomic absorption spectrometry analysis demonstrated that the differentiated SHSY5Y cells treated with 20 or 50 µM copper chloride can take up a greater quantity of metal. Copper uptake is mediated primarily by the specific transporters CTR1 and we showed that these copper treated cells increased the expression of the CTR1 transporter, further augmenting their metal uptake capacity. Once in the cell, small and soluble chaperone proteins are responsible for binding and delivery of copper to the intracellular compartments.

In line with a previous study by Hatori et al., we found that the CCS mRNA and the protein levels in the cells were not changed after copper incubation. CCS is a chaperone that transfers the metal to the antioxidant enzyme superoxide dismutase SOD1 [55] and its expression is regulated by copper itself. The CCS protein concentration is reduced in conditions of cell copper excess, probably by modeling its degradation by the proteasome machinery [56].

Here, we reported that, in the undifferentiated SHSY5Y cells treated with copper, the levels of total α-synuclein increased in a dose-dependent manner, while the correspondent phosphorylated form at S129 did not change. Conversely, a higher cell expression of the S129 phosphorylated form was present in differentiated cells after copper treatment, when compared to the untreated control conditions. In general, after the differentiation process, the SHSY5Y cells displayed a higher level of α-synuclein phosphorylated form, in comparison to the undifferentiated cells. These results could be explained by the augmented functional expression of the PLK2 kinase and by the unvaried levels of the correspondent PP2A phosphatase observed in the differentiated cells. PLK2 is a serine/threonine kinase that efficiently phosphorylates α-synuclein selectively on S129 residue [57]. The expression of this protein is significantly upregulated during aging and in the synucleinopathy-diseased brains [58]. Moreover, PLK2 binds the α-synuclein and enhances its autophagic degradation [59].

Based on previous research demonstrating the correlation between the oxidative stress, the phosphorylated state of the α-synuclein, and its consequent aggregation, we evaluated the cellular ROS amount in the cells. Contrary to our expectations, both the undifferentiated and differentiated cells treated with 20 or 50 µM copper for 48 h did not show altered cytosolic ROS levels or MMP. These results could explain our experimental observations regarding the absence of variation in expression levels of the Atox1 chaperone and the ATP7A transporter, after copper incubation. The Atox1 metallochaperone is responsible for shuttling the metal from the cytosol to the ATP7A and ATP7B transporters. The ATP7A is an ATPase transporter moving the copper to the trans-Golgi network or to the secretory vesicles pathway. The Atox1 was also shown to protect cells against the oxidative damage and it is upregulated by the ROS increase in neuronal cells [60,61].

In summary, we showed that the exposure of neuronal-like differentiated cells to copper for 48 h caused an accumulation of the phosphorylated α-synuclein, similar to what happens during the pathogenesis of the PD. Data obtained show that our cells do not suffer the oxidative stress due to the increase in intracellular copper following treatments. What we observed would appear to be linked to the overexpression of the PLK2 kinase, whose role in directing the α-synuclein protein towards the degradation pathway is well established [62,63]. However, the involvement of PP2A phosphatase cannot be completely ruled out and it would be worth further investigation. Even without altering its expression, as observed in our case, the α-synuclein-induced post translational modifications are known to seriously impair PP2A activity [64,65].

The disease occurrence in its sporadic form is known to depend on the disruption of certain pathways. The reference is generally made to the functional alterations of the mitochondria and the mitophagy mechanisms (with a consequent increased level of ROS in the cell) or to alterations in the proteostasis. However, given the high degree of integration between cellular processes, the appearance and persistence of one of these conditions is considered prodromal of the other.

What we observed may therefore represent one of the initial events in the onset of PD. We hypothesize that copper primarily determines an inhibition of the proteasome system leading to an accumulation of phosphorylated synuclein, with the oxidative stress occurring only at a later time.

Our findings advance the understanding of the correlation among copper and the formation of inclusion in PD and in the α-synucleinopathy disease pathogenesis.

## 4. Materials and Methods

### 4.1. Cell Cultures and Treatments

Human neuroblastoma SHSY5Y cells were purchased from European Collection of Cell Culture (Salisbury, UK) and grown in high-glucose Dulbecco’s Modified Eagle Medium (DMEM) (Sigma, St. Louis, MO) supplemented with 1 mM sodium pyruvate and 10% fetal calf serum (Sigma) at 37 °C in an atmosphere of 5% CO_2_. Neuronal differentiation of SHSY5Y was induced as previously described [27]. Briefly, cells were seeded at 3 × 10^4^ cells/cm^2^ onto a poly-lysine coated dish and sequentially treated with 10 µM RA and 2% FBS for 6 days, 10 µM RA (Sigma) and 1% FBS for 3 subsequent days and 50 ng/mL BDNF (PeproTech), 1X B27 (Gibco) in Neurobasal (Gibco) medium for the last 5 days. Differentiated and undifferentiated cells were treated with 1, 10, 20, 50, and 100 µM CuCl_2_ (Sigma) or 1, 5 and 10 µM rotenone (Sigma) for 24 or 48 h. Treatment of undifferentiated SHSY5Y cells was performed 24 h after plating by adding appropriate amounts of CuCl_2_ solution to the complete medium to avoid a possible overlapping toxicity due to serum deprivation.

### 4.2. Cell Viability Assay

Cell viability was evaluated by 3-(4,5-dimethylthiazol-2-yl)-5-(3-carboxymethylphenyl)-2-(4-sulphophenyl)-2H-tetrazolium MTT assay (Sigma) used to assess metabolic activity [66]. Cells were plated at a density of 1 × 10^4^ cells/well in 96-well plates and grown overnight. After exposure to copper or rotenone, cells were incubated with 50 µL of 0.5 mg/mL MTT for 3 h. The crystals of formazan were dissolved in dimethyl sulfoxide and absorbance (490 nm) was measured in a microplate reader (Thermo Fisher).

### 4.3. Analysis of Copper Content by Atomic Absorption

During atomic absorption spectrometry assays, multiple techniques can be used to dissolve organic samples before determining their metal content. A wet decomposition and oxidizing approach based on the use of nitric acid was chosen for our purpose. Cell mediums were collected, centrifugated (at 1200 rpm for 10 min) and treated with nitric acid at 0.5%. Cells were washed in PBS, collected, suspended in R/S buffer (7 M urea, 2 M thiourea, 4% CHAPS) for 20 min and finally centrifugated (13,000 rpm for 20 min). Cell lysates were diluted with nitric acid to achieve a 0.2% final concentration. After at least 2 weeks at room temperature, copper content was assayed using an Atomic Absorption Spectrometer (Varian AA-600Z) equipped with a dedicated graphite furnace model GTA-100 and Zeeman background correction. The detection wavelength was set at 327.4 nm.

### 4.4. Western Blot Analyses

Immunoelectrophoresis was performed on cell extracts obtained by treating cells with lysis buffer (Tris–HCl 10 mM, 150 mM NaCl, 1% Triton X-100, 1X Protease and Phosphatase Inhibitor Cocktail (Sigma)) for 20 min, followed by centrifugation (13,000 rpm, 20 min). Protein extracts were quantified with Bradford’s method, separated on 10% SDS–PAGE and then transferred on nitrocellulose membranes (Amersham Protean, GE Healthcare). Membranes were blocked for 1 h in Tris-buffered saline (TBS), 0.05% Tween-20, 5% non-fat dry milk, followed by overnight incubation with specific primary antibodies: anti-α-synuclein (Abcam, mouse mAb, 1:1000), anti-phospho-α-synuclein S129 (Abcam, rabbit mAb, 1:2000), anti-ATP7A (Novus, mouse mAb, 1:1000), anti-CTR1 (Novus, mouse mAb, 1:500), anti-MAP2 (Atlas Antibodies, mouse mAb, 1:1000), anti-CCS (Santa Cruz, mouse mAb, 1:1000), anti-PP2A (Millipore, mouse mAb, 1:5000), anti-actin (BioLegend, mouse mAb, 1:500). For the analysis of phospho-α-synuclein S129, the membrane was fixed in a 4% PFA, 0.1% glutaraldehyde PBS solution for 30 min before being blocking. Membranes were incubated for 1 h at room temperature with the secondary antibody (Sigma, goat anti-rabbit or anti-mouse horseradish peroxidase-linked IgG, 1:3000). Specific protein–antibody complexes were visualized with ECL Prime Western Blotting detection reagent (Amersham, GE Healthcare) and ChemiDoc System (Bio-Rad). Densitometric analysis were performed using Image Lab software (Bio-Rad) to determine optical density (OD) of the bands. The OD of phosphorylated proteins were expressed as a ratio relative to total protein (or to beta-actin). All other protein expressions were normalized to beta-actin to account for variations in loading.

### 4.5. Analysis of Mitochondrial Membrane Potential and ROS Production

The cationic JC-1 dye 5,5′,6,6′-tetrachloro-1,1′,3,3′-tetraethylbenzimidazolcarbo-cyanine iodide (Life Technologies) was used to analyze mitochondrial membrane potential [67]. It accumulates in energized mitochondria. At low concentrations, due to low mitochondrial membrane potential, JC-1 is predominantly a monomer that yields green fluorescence with an emission at 530 ± 15 nm.

At high concentrations, when trailed by an increased inner negative charge of the organelle, the dye aggregates yielding to a red to orange colored emission at a wavelength of 590 ± 17.5 nm. Therefore, the red/green fluorescence intensity ratio is indicative of the polarity and, therefore, of the functional state of the mitochondrion.

Briefly, the cells were seeded at 1.5 × 10^4^ cells/well in a 96-well microplate, grown overnight and treated as previously described. The cells were incubated with 2 µM JC-1 at 37 °C, 5% CO_2_, for 30 min and washed twice with PBS. The fluorescence was measured by a Synergy HTX microplate reader (BioTek): Ex/Em 488/535 nm for monomers and Ex/Em 488/590 nm for aggregates. After background subtraction (A590 of non-stained cells), ratio between monomer and aggregated forms measures was calculated.

The production of ROS was estimated using 2′,7′-dichlorofluorescin diacetate DCFDA (Sigma). Intracellular esterases cleave the molecule at the two ester bonds, producing the relatively polar cell membrane-impermeable product H2DCF; it accumulates intracellularly, and a subsequent oxidation, proportional to the concentration of hydrogen peroxide in the cells, yields the highly fluorescent product DCF. The fluorescence generated is directly proportional to the amount of oxidized DCFDA to DCF and can be measured in the green channel at about 529 nm. The redox state of the sample can be monitored by detecting the increase in fluorescence.

Briefly, the cells were seeded at 1.5 × 10^4^ cells/well in a 96-well microplate, grown overnight, and treated as previously described. Fresh medium containing DCFDA was added for 30 min. Cells were then washed with PBS and analyzed with a fluorescent microplate reader (Ex/Em 485/535 nm). The fluorescence generated is directly proportional to the amount of oxidized DCFDA to DCF.

### 4.6. Expression Analysis by qPCR

RNA was extracted from cells using Total RNA Purification Kit (Norgen Biotek Corp.). The quality and concentration of total RNA was determined with the NanoDrop ND-1000 Spectrophotometer (Thermo Scientific). Reverse transcription was performed on 500 ng of total RNA using PrimeScript cDNA synthesis mix (Takara), real-time PCR was executed with TB Green Premix Ex Taq II (Takara) and results determined with Rotor-Gene (QIAGEN). PCR primers for gene expression were designed with Primer3 software: SNCA (fw: 5′-ACCAAACAGGGTGTGGCAGAAG-3′, rev: 5′-CTTGCTCTTTGGTCTTCTCAGCC-3′), ATP7A (fw: 5′-ACCCTCTAGGAACAGCCATAACCA-3′, rev: 5′-ATACCACAGCCTGGCACAACCT-3′), CTR1 (fw: 5′-GACCAAATGGAACCATCCTT-3′, rev: 5′-ATGACCACCTGGATGATGTG-3′), synaptophysin (fw: 5′-ATTGTGCCAACAAGACCGAGAGT-3′, rev: 5′-CAGGAAGATGTAGGTGGCCAGAG-3′), ATOX1 (fw: 5′-TCTGAGCACAGCATGGACACTC -3′, rev: 5′-TCTGGAAGCCAGCGGGAGGAT-3′), COX17 (fw: 5′-TCTAATTGAGGCCCACAAGG-3′, rev: 5′-TCAGGAATTATTTATTCACACAGCA-3′), PLK2 (fw: 5′-CAACAATGGTGCTCACATGAGCC-3′, rev: 5′-GGAGCATCTGTTGCTGGGAAAAC-3′), PP2A (fw: 5′-GGTGGTCTCTCGCCATCTATAG-3′, rev: 5′-GGTGGTCTCTCGCCATCTATAG-3′), TH (fw: 5′-GCTGGACAAGTGTCATCACCTG-3′, rev: 5′-CCTGTACTGGAAGGCGATCTCA-3′). All reactions were performed in triplicate, including negative control samples, which never showed significant threshold cycles (*C_T_*). The relative expression of mRNAs was calculated by the 2^−ΔΔ*C*T^ method [68] using 18S ribosomal RNA as endogenous control ([*C_T(gene of interest)_* − *C_T(18S)_*] = Δ*C_T_*).

### 4.7. Statistical Analysis

The results are presented as means ± standard deviation from at least three biological replicates. Data were analyzed by using the one-way analysis of variance (ANOVA) with post-hoc Tukey test. Statistical analyses were performed and a value of *p* < 0.0332 was accepted as the level of significance (GraphPad Prism 8.0). The following statistical significance representations were used: * *p* < 0.0332 ** *p* < 0.0021 *** *p* < 0.0002.

## Data Availability

Data sharing not allowed.

## Abbreviations

Cu	copper
PD	Parkinson’s disease
CNS	central nervous system
ROS	reactive oxygen species
CTR1	copper transporter protein 1
PLK2	polo like kinase 2
PP2A	protein phosphatase 2A
LB	Lewy body
UPS	ubiquitin proteasome system
CTR2	copper transporter protein 2
Dmt1	divalent metal transporter 1
ATP7A	copper-transporting ATPases 1
ATP7B	copper-transporting ATPases 2
ATOX1	copper transport protein
SOD3	extracellular superoxide dismutase [Cu-Zn]
COX	cytochrome c oxidase
Cox17	cytochrome c oxidase copper chaperone
Sco1	cytochrome c oxidase assembly protein 1 chaperone
Sco2	cytochrome c oxidase assembly protein 2 chaperone
CCS	copper chaperone for superoxide dismutase
SOD1	superoxide dismutase [Cu-Zn]
TH	tyrosine hydroxylase
DAT	dopamine transporter
RA	retinoic acid
BDNF	brain-derived neurotrophic factor
MAP2	microtubule-associated protein 2
MTT	3-(4,5-dimethylthiazol-2-yl)-2,5-diphenyltetrazolium bromide
CTR	untreated cells
SNCA	α-synuclein gene
DCFDA	2′,7′-dichlorofluorescin diacetate
MMP	mitochondrial membrane potential
JC-1	5,5′,6,6′-tetrachloro-1,1′,3,3′-tetraethylbenzimidazolcarbo-cyanine iodide
DMEM	Dulbecco’s Modified Eagle Medium
MMP	mitochondrial membrane potential
MPT	membrane permeability transition
ANT	nucleotide translocase adenine
OD	optical density
TBS	Tris-buffered saline
